# Supplementary material for: Ajuforrestin A Inhibits Tumor Proliferation and Migration by Targeting the STAT3/FAK Signaling Pathways and VEGFR-2
Source: Biology (Basel). 2025 Jul 22;14(8):908. doi: 10.3390/biology14080908 (PMC12383317; doi:10.3390/biology14080908)
Supplement: Supplementary file 1 [file biology-14-00908-s001.zip › Biology-3614346-Supplementary data.pdf]

Supplementary data for

Ajuforrestin A Inhibits Tumor Proliferation and Migration by Targeting the STAT3/FAK Signaling Pathways and VEGFR-2

^1H and ^{13}C NMR spectra

Figure s1 ^1H NMR spectrum

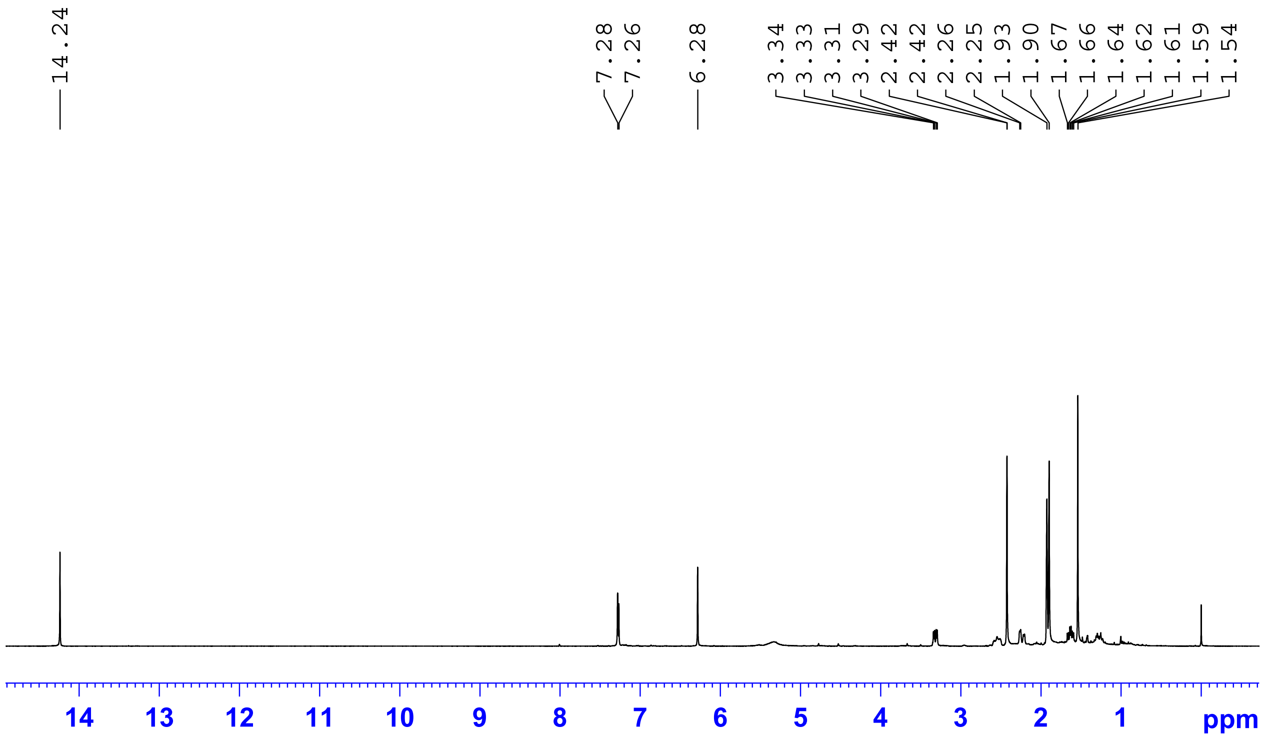

Figure s2 ^{13}C NMR spectrum

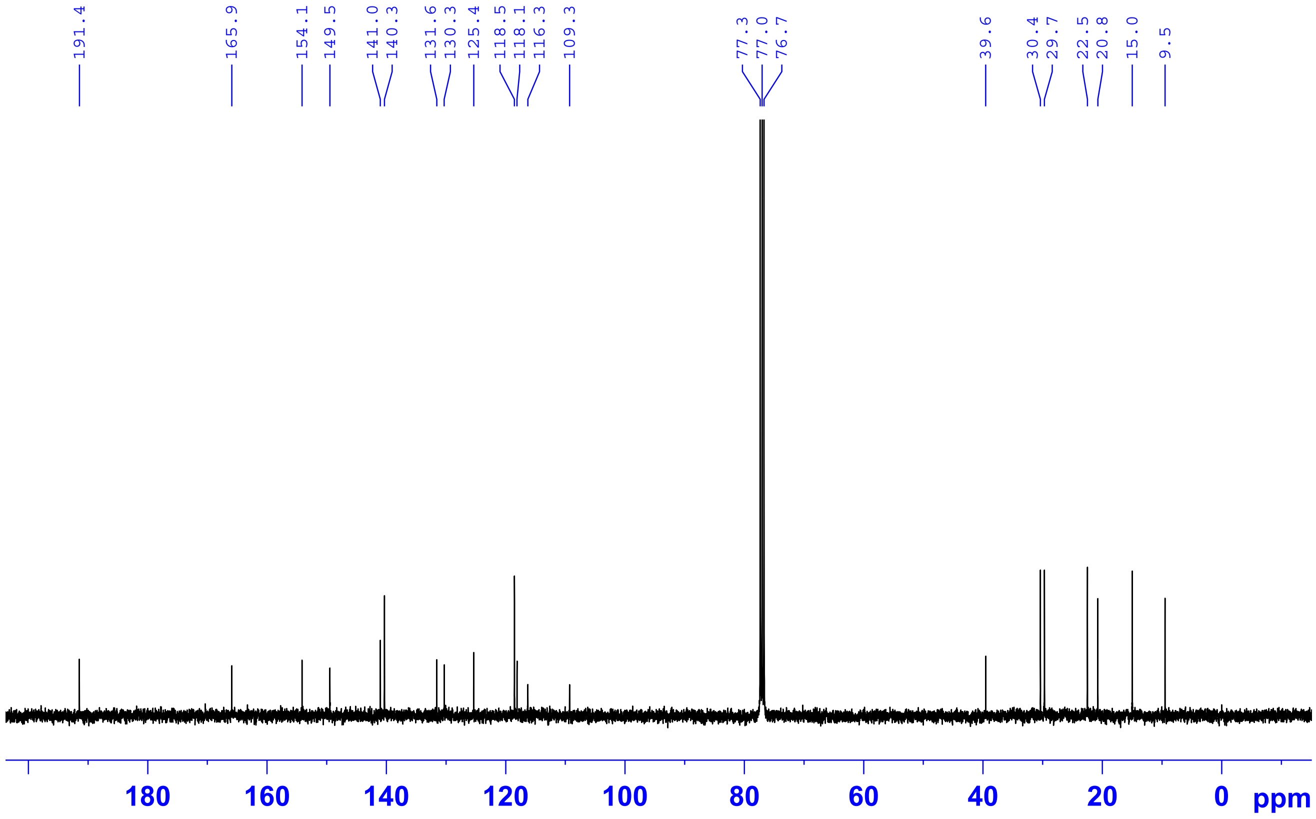

Materials and reagents

Fetal bovine serum (FBS) and Dulbecco's modified Eagle's Medium (DMEM) were obtained from LabBiotech Co., Ltd. (Jinan, China). MTT and DMSO were obtained from Solarbio Co., Ltd. (Beijing, China). Cell tracker CM-DiI was offered by Yeasen Biotechnology Co., Ltd. (Shanghai, China). The annexin V-FITC apoptosis detection kit, cell cycle and apoptosis kit, and BCA protein assay kit were purchased from Beyotime Biotechnology Co., Ltd. (Shanghai, China). All other reagents were of analytical grade and purchased from Tianjin Chemical Reagent Co. (Tianjin, China). The human lung adenocarcinoma cell line (A549) was obtained from the Cell Bank of the Chinese Academy of Sciences (Shanghai, China). Adult AB and transgenic zebrafish were provided by Shanghai Feixi Biotechnology Co., Ltd. (Shanghai, China).

In vitro cell viability assay

The MTT assay was utilized to evaluate the cytotoxic activity of ajuforrestin A on the A549 cell line [34]. Cells at the logarithmic growth stage were uniformly seeded into a 96-well plate (1×10^4 cells/well) and cultured at 37 °C for 24 h. Then, the cells were treated with different concentrations of ajuforrestin A and the positive control, etoposide. After 48 h treatment, 20 μL of MTT (5 mg/mL) solution was added to each well and incubated for another 4 h. The cells were centrifuged at 3000 rpm at room temperature for 20 min. After discarding the supernatant, 150 μL of DMSO was added to each well to dissolve the formazan crystals. After it was completely dissolved, the optical density (OD) value was measured at 492 nm by a microplate reader (Thermo Fisher Scientific Inc., Waltham, MA, USA). The experiment was carried out in triplicate.

Apoptosis analysis by flow cytometry

A549 cells were seeded into 12-well plates (1×10^5 cells /well) and placed in an incubator at 37°C and 5% CO_2. After 24 h of incubation, different concentrations of the tested compounds were added, and three paralleled wells were set up in every group. The adherent cells were rinsed with PBS twice, and then 195 μL of binding buffer was added. Then, the samples were stained with 5 μL Annexin V-FITC and 10 μL propyl iodide staining solution. After incubation at room temperature in the dark for 20 min, the cells were placed in an ice bath and shielded from light with aluminum foil. Then, flow cytometry was used to identify apoptosis [13,34]. Finally, the attained cells were detected by BD LSRFortessa flow cytometry (BD Biosciences, San Jose,

CA, USA). Flow cytometry data were analyzed with the FlowJo software (FlowJo LLC, Ashland, OR, USA).

Cell cycle analysis

The effects of ajuforrestin A on cell cycle distribution were assessed using flow cytometric analysis [35,36]. The cells in the logarithmic growth phase were seeded into 12-well plates (1 × 10^5 cells/well) and incubated at 37 °C for 24 h. Subsequently, the cells were treated with various concentrations of ajuforrestin A (5, 10, and 20 μM) for 48 h. Then, the cells were collected, washed twice with PBS, and fixed in 70% ice-cold ethanol at 4 °C for 24 h. The cells were then rinsed with PBS and stained with propidium iodide staining buffer containing RNase A at 37 ℃ in the dark for 30 min. The BD LSRFortessa flow cytometer was used to analyze the cellular DNA content, and the data were processed using ModFit LT software.

Wound-scratch assay

The inhibitory effects of ajuforrestin A on A549 tumor cell migration were determined by wound-healing assay [37]. The cells were collected and cultured in 6-well plates (5 × 10^5 cells/well) for 24 h. After reaching over 90% confluence, cells were subjected to scratching using a 200 μL sterile pipette tip. Then, the cell monolayers were rinsed with PBS to remove unattached cells and replenished with fresh culture solution. The fresh medium supplemented with various concentrations of ajuforrestin A was added. At 0 h and 48 h after the addition of the tested compounds, the cell monolayers were examined under a microscope and photographed. ImageJ software (NIH, Bethesda, Maryland, United States) was used to quantify scratch area and calculate mobility.

Western blotting analysis

A549 cells were inoculated into 6-well plates (1 × 10^5 cells/well) for 24 h. Subsequently, the cells were administered with ajuforrestin A for another 48 h. The cells were rinsed twice with cold PBS and subsequently lysed using RIPA lysis solution (Beyotime, P0013B) at 4 ℃ for 30 min. The cells were centrifuged for 10 min at 10,000 rpm, and the supernatant was harvested to isolate the total proteins. The protein concentration was measured with the BCA protein assay kit (Beyotime, P0012S). Protein samples were fractionated using gel

electrophoresis (SDS-PAGE) and subsequently electrotransferred on polyvinylidene difluoride (PVDF) membranes. Membranes were incubated with 5% skim milk in TBST solution at room temperature for 1 h, followed by overnight incubation at 4 °C with the appropriate primary antibodies. Following TBST washing, the membranes were incubated with horseradish peroxidase-conjugated secondary antibodies for 1 hour at room temperature, and subsequently washed three times with TBST. An ECL luminous solution was applied and imaged using a chemiluminescent imager, with the bands quantitatively evaluated via ImageJ software.

Zebrafish husbandry and maintenance

Zebrafish were cultivated in a recirculating aquatic system with aeration and ultraviolet sterilizing. The zebrafish were maintained in a 14-hour light/10-hour dark cycle at 28.5 ℃ and fed with brine shrimp twice daily. Zebrafish embryos were obtained from mating adults according to previously described procedures [34,36]. All animal-related operations received approval from the Institutional Animal Ethics Committee of Nankai University (2021-SYDWLL-000225).

Antiangiogenetic assay using a transgenic zebrafish model

Zebrafish embryos were obtained by mating adult zebrafish using *Tg* (*fli1:EGFP*) transgenic zebrafish [38,39]. Embryos of 6 h post-fertilization (hpf) were randomly transferred to 12-well plates (15 embryos/group). The tested compounds were then added and incubated at 28.5°C for 48 hours. After the treatment, the development of intersegmental vessels (ISVs) and dorsal longitudinal anastomosing vessels (DLAVs) was visualized by confocal microscopy (Leica, TCS SP8, Germany), and the length of the ISVs was quantified using ImageJ software.

Surface plasmon resonance assay

The binding kinetics between ajuforrestin A and VEGFR-2 were investigated by surface plasmon resonance using a Biacore T200 instrument (GE Healthcare, Stockholm, Sweden) [40,41]. VEGFR-2 was immobilized on a CM5 sensor chip using amine-coupling chemistry as described by the manufacturer. For the measurement of interactions between VEGFR-2 and ajuforrestin A, a continuous flow (30 μL/min) of serially diluted

ajuforrestin A (1.56, 3.12, 6.25, 12.5, 25, 50, 100, and 200 μM) onto the immobilized ligand surface was monitored by passing the analytes across the sensor chip. All processes were conducted in HBSEP + running buffer (pH 7.4). Binding data was assessed using Biacore T200 Evaluation Software (Version 1.0, GE Healthcare, Stockholm, Sweden).

***In vivo* antitumor assay using zebrafish tumor xenografts**

The xenograft tumor model was constructed as previously reported [39,42]. Firstly, the fluorescently labeled tumor cells were prepared, the cells were collected, centrifuged, and resuspended, and the CM-DiI staining solution at a final concentration of 2 μM was added. After washing twice with PBS, the stained cells were resuspended in serum-free DMEM medium at a density of 1×10^7 cells/mL. Then, normal and healthy embryos were randomly selected to be anesthetized with tricaine, and then fixed and placed neatly with 2% sodium carboxymethyl cellulose. 5 nL of the fluorescently labeled tumor cells prepared above were microinjected into the yolk sac of 48 hpf embryos successively with a microinjection system. After incubation for 6 h, the embryos were randomly divided into groups (15 embryos/group) and treated with various concentrations of ajuforrestin A (5, 10, and 20 μM) and the positive control etoposide (10 μM) for 48 h. The embryos were then anesthetized with 0.02% tricaine and imaged using a confocal microscope (Leica, TCS SP8, Germany). The density and focus number of red fluorescence were quantified by ImageJ software.
