# Supplementary figures and images for: Ajuforrestin A Inhibits Tumor Proliferation and Migration by Targeting the STAT3/FAK Signaling Pathways and VEGFR-2
Source: Biology (Basel). 2025 Jul 22;14(8):908. doi: 10.3390/biology14080908 (PMC12383317; doi:10.3390/biology14080908)

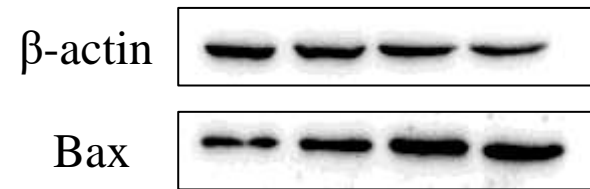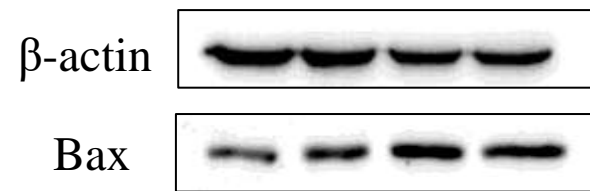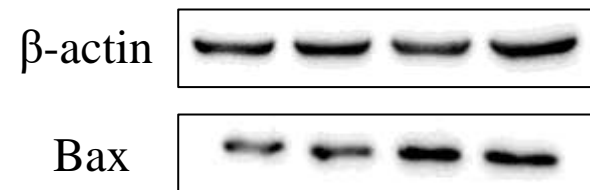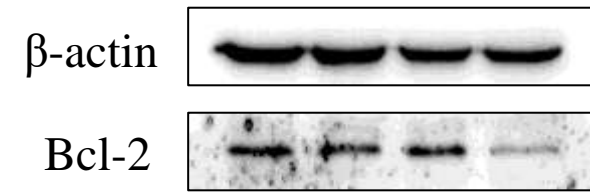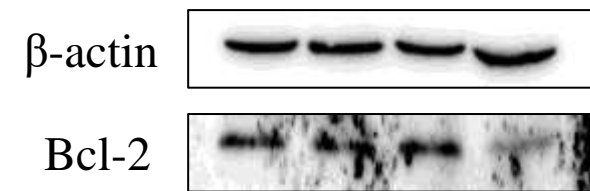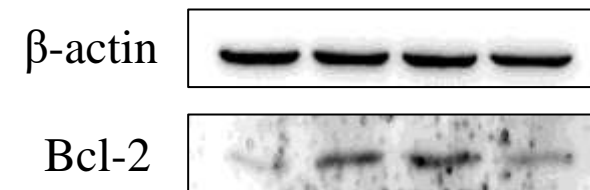

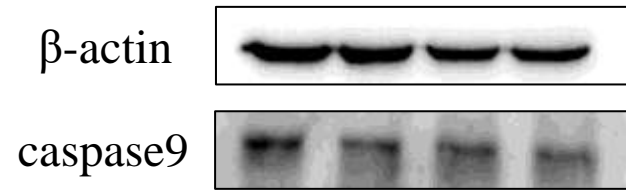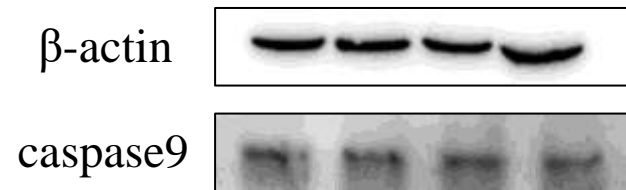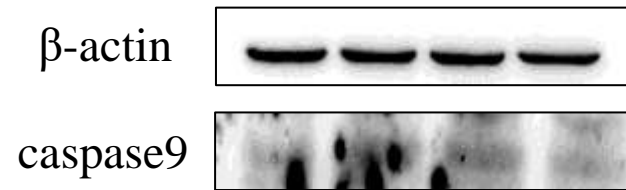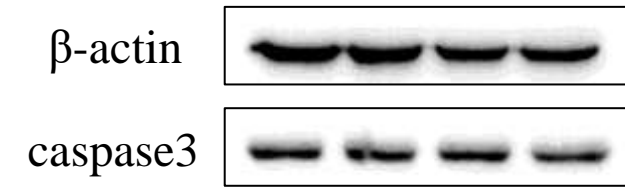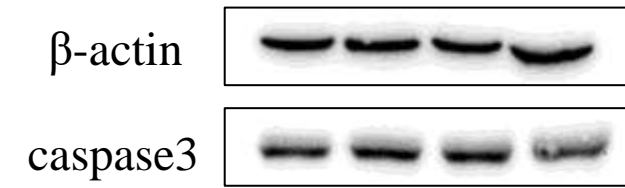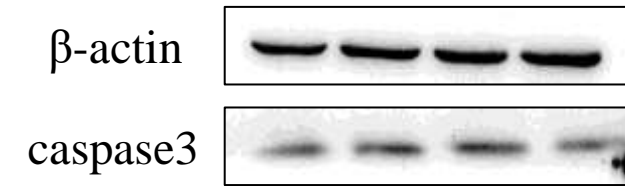

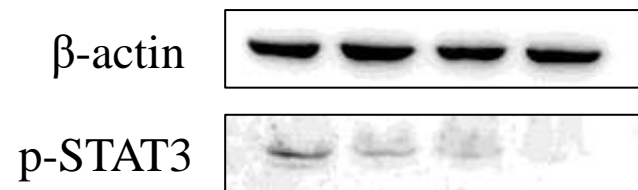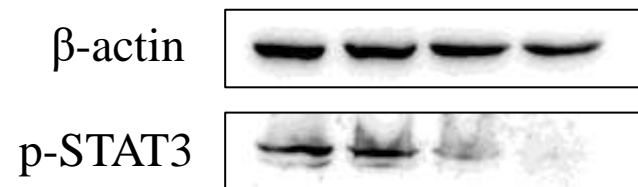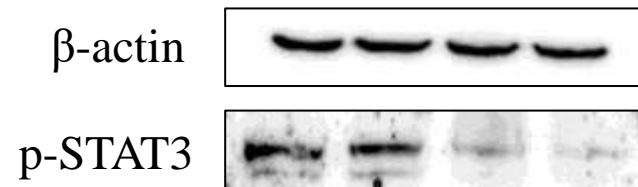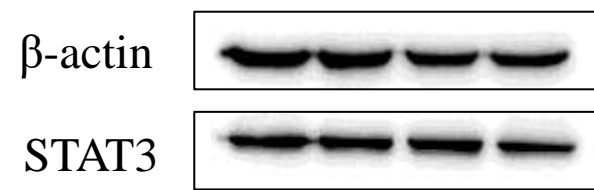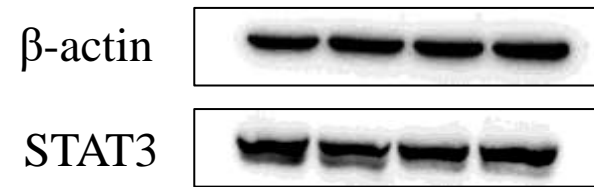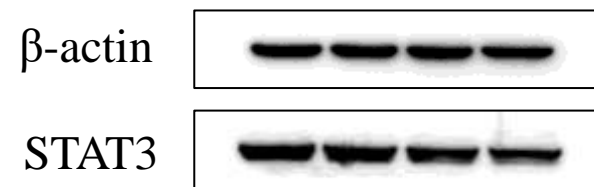

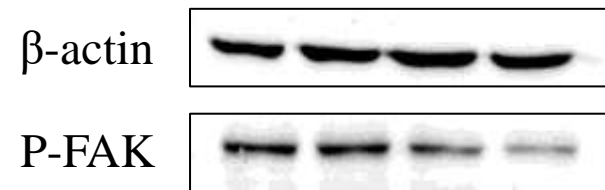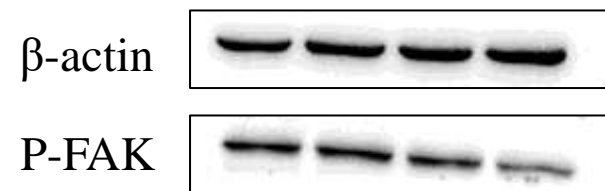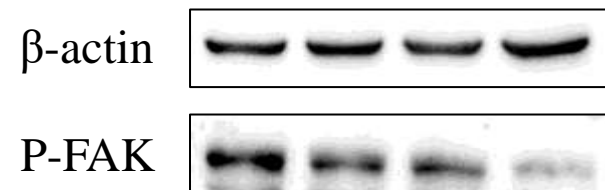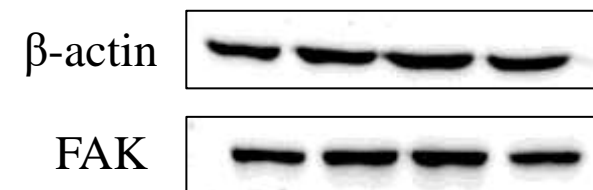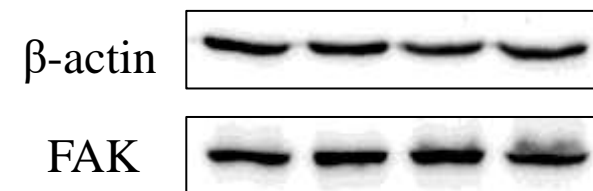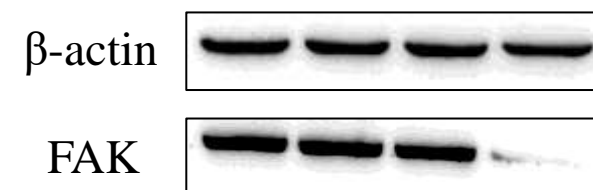

$\beta$ -actin

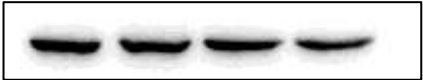

MMP2

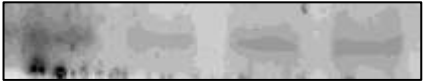

$\beta$ -actin

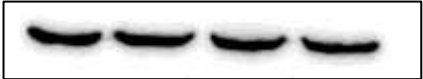

MMP2

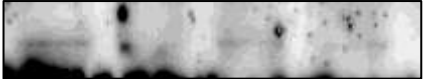

$\beta$ -actin

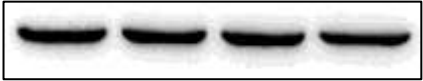

MMP2

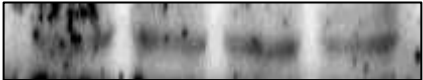

Supplement: Supplementary file 1 [file biology-14-00908-s001.zip › Biology-3614346-Western blotting figures.pdf]
